# CSF1R-Expressing Tumor-Associated Macrophages, Smoking and Survival in Lung Adenocarcinoma: Analyses Using Quantitative Phosphor-Integrated Dot Staining

**DOI:** 10.3390/cancers10080252

**Published:** 2018-07-31

**Authors:** Kentaro Inamura, Yasuyuki Shigematsu, Hironori Ninomiya, Yasuhiro Nakashima, Maki Kobayashi, Haruyuki Saito, Katsuhiro Takahashi, Etsuko Futaya, Sakae Okumura, Yuichi Ishikawa, Hiroaki Kanda

**Affiliations:** 1 Division of Pathology, The Cancer Institute, Department of Pathology, The Cancer Institute Hospital, Japanese Foundation for Cancer Research, 3-8-31 Ariake, Koto-ku, Tokyo 135-8550, Japan; yasuyuki.shigematsu@jfcr.or.jp (Y.S.); hironori.ninomiya@jfcr.or.jp (H.N.); midysland@gmail.com (Y.N.); maki.kobayashi@jfcr.or.jp (M.K.); ishikawa@jfcr.or.jp (Y.I.); hkanda@cancer-c.pref.saitama.jp (H.K.); 2 Department of Thoracic Surgical Oncology, The Cancer Institute Hospital, Japanese Foundation for Cancer Research, 3-8-31 Ariake, Koto-ku, Tokyo 135-8550, Japan; sokumura@jfcr.or.jp; 3 Bio Advanced Technology Division, Corporate R&D Headquarters, Konica Minolta, Inc., 1 Sakura-machi, Hino, Tokyo 191-8511, Japan; haruyuki.saito@konicaminolta.com (H.S.); katsuhiro.takahashi@konicaminolta.com (K.T.); etsuko.futaya@konicaminolta.com (E.F.); 4 Department of Pathology, Saitama Cancer Center, 780 Komuro, Ina, Kitaadachi-gun, Saitama 362-0806, Japan

**Keywords:** CSF1R, tumor-associated macrophage (TAM), phosphor-integrated dot (PID), tobacco, non-small-cell lung cancer (NSCLC), immunohistochemistry, neoantigens, tumor mutational burden, immune checkpoint inhibitor, CD163

## Abstract

CSF1R-expressing tumor-associated macrophages (TAMs) induce a tumor-promoting microenvironment by regulating immunity. Evidence demonstrates that the expression and single nucleotide polymorphisms of *CSF1R* relate with survival and risk of lung cancer in never smokers. However, no previous studies have examined the association of CSF1R expression in TAMs with mortality or whether the prognostic association differs according to smoking status in lung adenocarcinoma. Quantitative phosphor-integrated dot staining was used to precisely assess CSF1R expression in TAMs. Using 195 consecutive cases of lung adenocarcinoma, we examined the association of CSF1R expression with mortality and whether the prognostic association differs according to smoking status. We observed high expression levels of CSF1R in TAMs in 65 of 195 (33%) cases of lung adenocarcinoma. High expression levels of CSF1R were associated with high lung cancer-specific mortality (log-rank *p* = 0.037; hazard ratio (HR) = 1.61, 95% confidence interval (CI) = 1.02−2.52, *p* = 0.043). This prognostic association differed according to smoking status (*p* for interaction = 0.049, between never-smoking and ever-smoking patients). The association between high expression levels of CSF1R and lung cancer-specific mortality was stronger in never-smoking patients (log-rank *p* = 0.0027; HR = 2.90, 95% CI = 1.41−6.11, *p* = 0.0041) than in ever-smoking patients (log-rank *p* = 0.73; HR = 1.11, 95% CI = 0.59−2.00, *p* = 0.73). The findings suggest that CSF1R-expressing TAMs may exert stronger tumor-promoting immunity in never-smoking patients with lung adenocarcinoma and serve as a therapeutic target in precision immunotherapies.

## 1. Introduction

Lung adenocarcinomas exhibit molecular features that differ according to the smoking history of the patient. Tobacco smoking creates DNA damage and induces neoantigens, which are important targets for antitumor immunity after administration of immune checkpoint inhibitors. Lung adenocarcinoma in smokers is characterized by a tobacco-induced mutational burden and pro-inflammatory tumor microenvironment, which may explain its responsiveness to treatment with immunotherapies [1,2,3]. In contrast, lung adenocarcinoma in never-smokers harbors a low mutational burden and immunosuppressive tumor microenvironment, which inactivates antitumor immunity and may lead to immunotherapy refractoriness [1,2,3]. Emerging evidence has demonstrated that the tumor microenvironment is differentially regulated by specific immune modulators that differ according to the smoking history of the patient [4,5,6,7].

CSF1R is a receptor tyrosine kinase that mediates tumorigenesis in tumor-immune microenvironments and is expressed on tumor-associated macrophages (TAMs) [8,9,10]; the high expression levels of CSF1R correlate with poor survival in patients with various malignancies [8,9,10,11]. Recently, CSF1R-targeted therapies have emerged as a promising new class of immune-modulatory drugs [8,9,10,12,13,14]. Evidence demonstrates that the expression and single nucleotide polymorphisms (SNPs) of *CSF1R* relate with survival and risk of lung cancer in never-smokers [15,16,17]; however, no studies have examined the association of CSF1R expression in TAMs with mortality or the prognostic interaction between CSF1R expression and smoking in lung adenocarcinoma. Elucidation of the prognostic association would inform future researches examining the role of CSF1R and the potential interplay of CSF1R expression in TAMs and smoking status.

Therefore, we examined the association between high expression levels of CSF1R in TAMs and patient mortality and assessed whether the prognostic association differs according to smoking status, using 195 consecutive cases of lung adenocarcinoma. Quantitative phosphor-integrated dot (PID) staining was used to precisely assess the expression levels of the CSF1R protein in TAMs [18,19].

## 2. Materials and Methods

### 2.1. Patients

On the basis of the availability of patient data on CSF1R expression status in TAMs, smoking status and survival, we enrolled 195 consecutive Japanese patients with lung adenocarcinoma who had undergone surgical resection between April 1995 and January 2002 at The Cancer Institute Hospital, Japanese Foundation for Cancer Research (JFCR), Tokyo, Japan [20,21,22]. Patients were observed until death or December 2016, whichever came first. Smoking histories were obtained from rigorous interviews of each patient by experienced thoracic surgeons. Pathological diagnoses were made by experienced pulmonary pathologists (Kentaro Inamura and Yuichi Ishikawa) basically according to the 2015 WHO classification of lung tumors [23]. All patients were pathologically staged according to the AJCC-TNM staging system, 7th edition [24]. The study protocol was approved by the International Review Board of JFCR on 27 October 2017 (ethic code: 2017-1085), and informed consent was obtained from all patients.

### 2.2. Immunostaining

Sequential triple immunostaining was carried out on previously constructed tissue microarrays [20,21,22] for CD68 [diaminobenzidine (DAB) staining], CD163 (HistoGreen staining), and CSF1R (PID staining). Four-micron-thick sections were deparaffinized and immersed in antigen retrieval solution (10 mM Tris buffer, pH 9) for 45 min at 95 °C. Endogenous peroxidase activity was blocked by treatment with 3% hydrogen peroxide for 15 min, followed by blockade of nonspecific reactions by immersion in phosphate-buffered saline (PBS) containing 1% bovine serum albumin (BSA) for 15 min. The sections were incubated with anti-CD68 mouse monoclonal antibody (1:100; clone: PG-M1, Dako, Glostrup, Denmark) for 60 min at 25 °C, followed by treatment with the Universal Immunoperoxidase Polymer (Nichirei Biosciences, Tokyo, Japan) for 30 min at 25 °C. The sections were then visualized with DAB (Wako, Osaka, Japan) for 3 min at room temperature. After a second round of antigen retrieval and blockade of nonspecific reactions, the sections were incubated with anti-CD163 mouse monoclonal antibody (1:50; clone: 10D6, Abcam, Cambridge, UK) overnight at 4 °C, followed by treatment with the Universal Immunoperoxidase Polymer for 30 min at 25 °C. The sections were visualized with the addition of HistoGreen (AbCys, Paris, France) for 3 min at room temperature, followed by washing in PBS and distilled water. After blocking nonspecific reactions, the sections were incubated with anti-CSF1R rabbit monoclonal antibody (1:50; clone: SP211, Abcam) overnight at 4 °C, followed by treatment with biotinylated anti-rabbit monoclonal antibody (clone: LO-RG-1, Bio-Rad, Hercules, CA, USA) for 30 min at 25 °C. The sections were then visualized with PID-conjugated streptavidin (0.09 nM) for 120 min at 25 °C. After washing in PBS, the sections were fixed with 4% paraformaldehyde and immersed in hematoxylin for counterstaining. SK-BR3 cells (ATCC, Manassas, VA, USA), which express CSF1R, and human lymph nodes, which express CD68 and CD163, were used as positive controls. Sections processed with replacement of primary anti-CSF1R antibody with PBS containing 1% BSA were used as negative controls. To assess the heterogeneity of CSF1R expression in TAMs, we immunohistochemically stained 10 cases of lung adenocarcinoma for CSF1R, CD68 and CD163; we did not observe substantial intratumoral or peritumoral heterogeneity in terms of CSF1R expression in TAMs.

### 2.3. Measurement of CSF1R Expression in TAMs

TAMs were defined as intratumoral or peritumoral cells that stained positive for both CD68 (cytoplasmic DAB staining) and CD163 (membranous HistoGreen staining). An entire image of each case was acquired using an Aperio image analysis system (Leica Biosystems, New Castle, UK). In each case, bright- and dark-field images were taken in at least five fields [19,25] (with 196 × 147 µm in size) using a fluorescence microscope (BX63, Olympus, Tokyo, Japan) connected to a DP80 CCD camera (Olympus). In each field, the number of TAMs was counted, and the number of PID particles per TAM was measured using a software for analyzing PID (PID analyzer, Konica Minolta, Tokyo, Japan), as described previously [18]. Five fields were randomly selected, and the numbers of TAMs and PID particles per TAM in the five fields were averaged and used to score each case. The upper tertiles of the average numbers of TAMs and PID particles per TAM were defined as a high number of TAMs and a high expression level of CSF1R in each case, respectively. The lower and middle tertiles were defined as a low–moderate number of TAMs and a low–moderate expression level of CSF1R in each case, respectively.

### 2.4. Detection of EGFR and KRAS Mutations and ALK Fusions

Tumor specimens were snap-frozen in liquid nitrogen within 20 min of surgical removal and stored at −80 °C until use. DNA was extracted by standard proteinase K digestion and phenol–chloroform extraction. For analysis of *EGFR* mutations, we examined four exons (exons 18–21) that encode the tyrosine kinase domain of the *EGFR* gene. For exons 18 (G719X), 20 (S768I and T790M), and 21 (L858R and L861Q), the TaqMan^TM^ SNP Genotyping Assay (Applied Biosystems, Foster City, CA, USA) was performed, according to the manufacturer’s instructions. Fragment analysis was conducted for the exon 19 deletion and the exon 20 insertion, as described previously [20,21]. To analyze *KRAS* mutations, we directly sequenced codons 12, 13 and 61, as described previously [20,21]. To detect *ALK* fusions, we performed ALK immunohistochemistry using an anti-ALK mouse monoclonal antibody (1:50; clone: 5A4, Leica Biosystems Newcastle Ltd., Newcastle, UK) and the Leica Bond III automated system (Leica Biosystems Melbourne Pty Ltd., Melbourne, Australia). The sections were incubated at pH 9 for 30 min at 100 °C. All fusions in the ALK-positive cases were confirmed by fluorescence in situ hybridization, as described previously [26].

### 2.5. Statistical Analysis

All statistical analyses were conducted using JMP 12 software (SAS Institute Inc., Cary, NC, USA). All two-sided *p* values less than 0.05 were considered statistically significant. To investigate the association of CSF1R expression status in TAMs (low–moderate vs. high) with clinicopathological and molecular features, we performed Chi-square or Fisher’s exact test appropriately.

For survival analyses, we used the Kaplan–Meier method and log-rank test. Survival time was defined as the duration from the date of surgery to death or the end of follow-up. In lung cancer-specific survival analysis, deaths as a result of other causes were censored. Cox proportional hazards regression models were used to calculate hazard ratios (HRs) and 95% confidence intervals (CIs) for mortality, according to CSF1R expression status (low–moderate vs. high). In addition to CSF1R expression status, the multivariable model included age at surgery, gender, smoking status, pathological stage, tumor differentiation grade, *EGFR* status, *KRAS* status, *ALK* rearrangement and number of TAMs. A backward stepwise elimination with *p* equal to 0.05 as the threshold was performed to select variables for the final models. *P* values for interactions between CSF1R expression status and smoking status were assessed using the Wald test on the cross-product of the CSF1R expression status (low–moderate vs. high) and smoking status variables (never- vs. ever-smoker) in the Cox model.

## 3. Results

### 3.1. CSF1R Expression in TAMs

**Figure 1:**
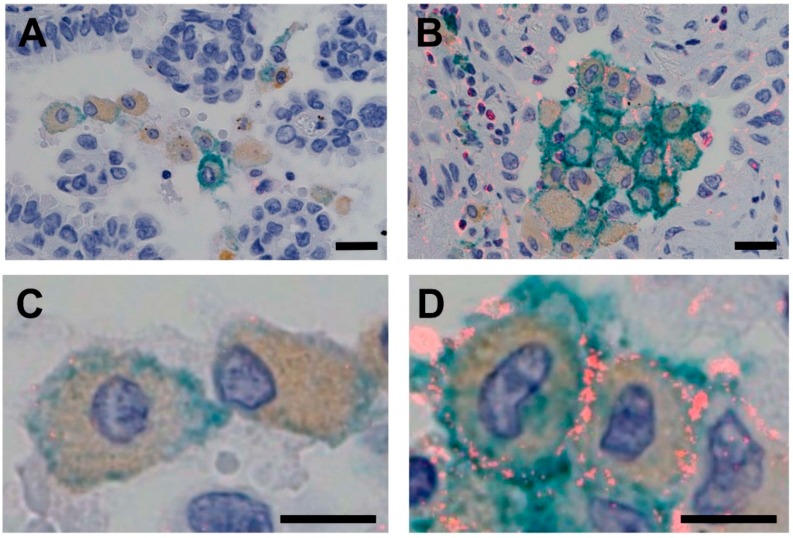
Triple-stained images for PID, DAB and HistoGreen. CSF1R-expressing TAMs stained positive for CSF1R (red), CD68 (brown) and CD163 (green). (1) TAMs with low expression levels of CSF1R (**A**: low magnification, scale bar = 20 µm; **C**: high magnification, scale bar = 10 µm). (2) TAMs with high expression levels of CSF1R (**B**: low magnification, scale bar = 20 µm; **D**: high magnification, scale bar = 10 µm). DAB, diaminobenzidine; PID, phosphor-integrated dot; TAM, tumor-associated macrophage.

Of the 195 cases of lung adenocarcinoma, we observed 65 cases (33%) in which CSF1R expression in TAMs was high using PID immunohistochemistry (Figure 1). Table 1 summarizes the clinicopathological and molecular characteristics of cases of lung adenocarcinoma, according to CSF1R expression status (low–moderate vs. high). High expression levels of CSF1R were associated with a less-differentiated grade of adenocarcinoma (*p* = 0.012).

### 3.2. Association of CSF1R Expression in TAMs with Survival in Patients with Lung Adenocarcinoma

**Figure 2:**
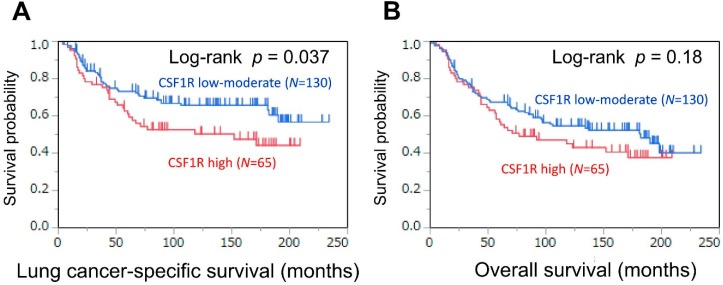
Kaplan–Meier curves for lung cancer-specific (**A**) and overall (**B**) survival, according to CSF1R expression status of tumor-associated macrophages (low–moderate vs. high), in lung adenocarcinoma patients.

There were 101 deaths, including 77 lung cancer-specific deaths, during a median follow-up period of 134 months (interquartile range: 37–168 months) of 195 patients with lung adenocarcinoma. The 5 years lung cancer-specific survival and overall survival rates were 69% and 64%, respectively. We assessed the association between CSF1R expression in TAMs and survival (Figure 2 and Table 2). In a Kaplan–Meier analysis, high expression levels of CSF1R were associated with higher lung cancer-specific mortality (5 years survival: 61 months) than low–moderate expression levels of CSF1R (5 years survival: 73 months; log-rank *p* = 0.037) (Figure 2A). In a Cox regression analysis, high expression levels of CSF1R were associated with high lung cancer-specific mortality in both univariable (HR = 1.61, 95% CI = 1.02−2.52; *p* = 0.043) and multivariable analyses (HR = 1.32, 95% CI = 1.00−2.49, *p* = 0.048) (Table 2)

### 3.3. Association of CSF1R Expression in TAMs with Survival, Stratified by Smoking Status

**Figure 3:**
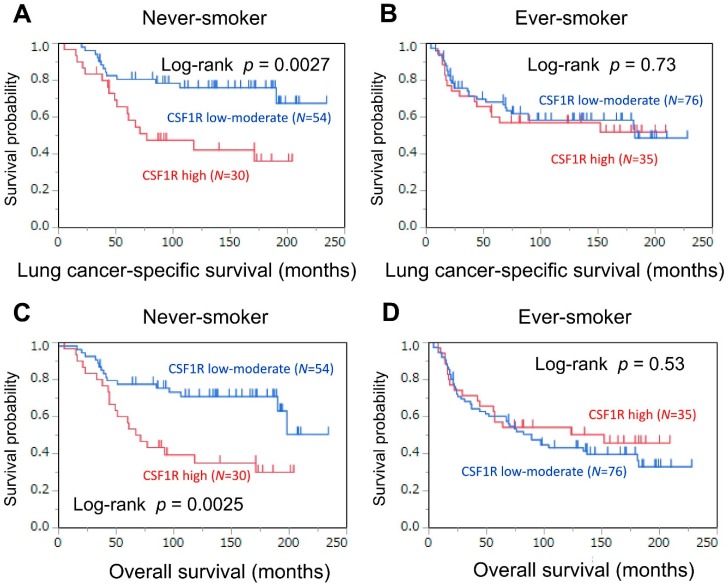
Kaplan–Meier curves for lung cancer-specific (**A**) and overall (**C**) survival, according to CSF1R expression status in tumor-associated macrophages (low–moderate vs. high), in never-smoking patients. Kaplan–Meier curves for lung cancer-specific (**B**) and overall (**D**) survival, according to CSF1R expression status in tumor-associated macrophages (low–moderate vs. high), in ever-smoking patients.

We examined whether the association of CSF1R expression in TAMs with mortality differed according to smoking status (Table 2 and Figure 3). High expression levels of CSF1R were associated with high lung cancer-specific mortality (log-rank *p* = 0.0027; Figure 3A) in never-smoking patients; however, high expression levels of CSF1R were not associated with lung cancer-specific mortality (log-rank *p* = 0.73; Figure 3B) in ever-smoking patients. In a Cox regression analysis (Table 2), high expression levels of CSF1R were associated with high lung cancer-specific mortality in both univariable (HR = 2.90, 95% CI = 1.41−6.11, *p* = 0.0041) and multivariable analyses (HR = 2.66, 95% CI = 1.28−5.66, *p* = 0.0088) in never-smoking patients; however, such associations were not observed in univariable (*p* = 0.73) or multivariable analyses (*p* = 0.54) in ever-smoking patients. In a multivariable Cox model, the *P* value for a prognostic interaction between CSF1R expression (low–moderate vs. high) and smoking status (never- vs. ever-smoker) was not significant (*P* for interaction = 0.12) after adjusting for the pathological stage of the adenocarcinoma. However, in a univariable Cox model, there was a significant prognostic interaction (*p* for interaction = 0.049) between CSF1R expression and smoking status (Table 2).

### 3.4. Association of CSF1R Expression in TAMs with Survival in Female Patients, Stratified by Smoking Status

**Figure 4:**
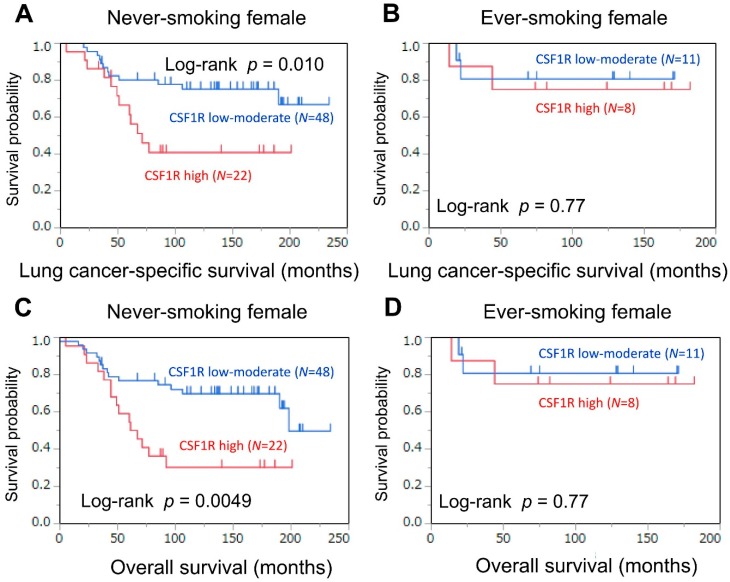
Kaplan–Meier curves for lung cancer-specific (**A**) and overall (**C**) survival, according to CSF1R expression status in tumor-associated macrophages (low–moderate vs. high), in never-smoking female patients. Kaplan–Meier curves for lung cancer-specific (**B**) and overall (**D**) survival, according to CSF1R expression status in tumor-associated macrophages (low–moderate vs. high), in ever-smoking female patients.

As an exploratory analysis, we assessed the association of CSF1R expression with mortality in female patients, stratified by smoking status (Table 3 and Figure 4), because *CSF1R* SNPs have been associated with both risk of lung cancer and survival in never-smoking females [15,16]. In never-smoking female patients, high expression levels of CSF1R were associated with high lung cancer-specific mortality (log-rank *p* = 0.010; univariable HR = 2.78, 95% CI = 1.22−6.32, *p* = 0.015). In contrast, such a prognostic association was not observed in ever-smoking female patients (log-rank *p* = 0.77). The *p* values for prognostic interactions between CSF1R expression and smoking status were not significant (*p* values for interaction >0.29), although the statistical power was limited in this subgroup analysis.

## 4. Discussion

CSF1R-expressing TAMs induce a tumor-promoting microenvironment by regulating immunity. Evidence demonstrates that the expression and SNPs of *CSF1R* are associated with survival and risk of lung cancer in never smokers [15,16,17]; however, the association of CSF1R expression in TAMs with mortality and the prognostic interaction between CSF1R expression and smoking status have not been previously examined. Therefore, we conducted this study to examine the association of CSF1R expression in TAMs with mortality and determine whether this association differs according to smoking status in cases of lung adenocarcinoma using PID immunostaining. We found that high expression levels of CSF1R were associated with higher mortality in never-smoking patients compared with ever-smoking patients. Our results provide evidence for a potential interaction between CSF1R expression in TAMs and smoking status in the progression of lung adenocarcinoma. Our findings, if validated, would inform future researches examining the interplay of CSF1R expression in TAMs and smoking status.

Lung adenocarcinoma represents a group of clinicopathologically and molecularly heterogeneous diseases [23,27,28,29,30,31,32,33,34,35,36,37,38]. A history of smoking substantially affects the molecular features of these tumors. Lung adenocarcinomas in smokers, which are relatively susceptible to immune checkpoint inhibitors, exhibit a smoking-specific mutational signature, high mutational load, and pro-inflammatory tumor microenvironment [1,2,3]. In contrast, lung adenocarcinomas in never-smokers, which are often refractory to these immunotherapies, harbor less genomic complexity, lower mutational load, and immunosuppressive tumor microenvironment [1,2,3]. Emerging evidence has demonstrated that substantial differences exist in the lung adenocarcinoma microenvironment between smokers and non-smokers [4,5,6,7]. The tumor microenvironment appears to be differentially orchestrated by specific immune modulators in the context of smoking status [4,5,6,7].

CSF1R-expressing TAMs promote self-maintenance functions and tumorigenic processes, such as escape from immune surveillance [10,12]. Observational studies have demonstrated an association between high expression levels of CSF1R and poor survival in patients with various malignancies [8,9,10,11]. As a receptor tyrosine kinase, CSF1R is an attractive therapeutic target, considering the tumor-permissive and immunosuppressive characteristics of CSF1R-expressing TAMs. A variety of small molecules and monoclonal antibodies targeting CSF1R are in clinical development as monotherapies and combination therapies with chemotherapies or other immunotherapies. Given patient tolerance of CSF1R-targeted therapies, CSF1R inhibitors have emerged as a promising new class of immune-modulatory drugs [8,9,10,12,13,14]. In the current study, high expression levels of CSF1R were associated with high mortality in patients with lung adenocarcinoma; this prognostic association was stronger in never-smoking patients than in ever-smoking patients. These findings suggest that CSF1R-expressing TAMs may induce a tumor-promoting microenvironment, especially in never-smoking patients with lung adenocarcinoma. Never-smoking patients may thus be potential candidates for CSF1R-targeted therapies.

TAMs exhibit both anti-tumor and tumor-promoting functions, depending on their acquired immunophenotype (M1 or M2) [39,40,41,42]. M2-TAMs, which induce a tumor-promoting microenvironment, are characterized by co-expression of CD68 and CD163. High expression levels of CD163 in macrophages relate with high mortality in malignancies, including lung adenocarcinoma [39,40,41,42]. Furthermore, high expression levels of CD163 have been associated with high expression levels of CSF1, which is one of the ligands of CSF1R [41]. The association between expression levels of CD163 and CSF1R is intriguing, and thus, needs to be investigated.

Accumulating evidence indicates that tumor molecular alterations are associated with infiltration of specific immune-cell subtypes in tumor microenvironment [43,44,45]. This evidence supports the hypothesis that tumor cells may orchestrate their immune microenvironment [43]. In lung adenocarcinoma, *EGFR* wild-type tumors have been characterized by higher density of neutrophils and macrophages [43]. In the current study, expression levels of CSF1R in TAMs were not associated with driver genetic alterations. Further research is required to confirm our results.

The quantitative detection of PID nanoparticles allowed us to precisely measure the expression levels of CSF1R protein in TAMs. Although conventional immunohistochemistry with DAB is widely used, it has several limitations. The intensity of DAB staining depends on enzymatic activity and is substantially influenced by incubation time/temperature and signal enhancement [46]. Because of these factors, the sensitivity of immunohistochemistry with DAB is low [18]. Furthermore, co-localized proteins cannot be distinguished by the chromogenic method. In contrast, the PID method enables us to distinguish the PID-stained protein from the co-localized protein with chromogenic staining. Fluorescent immunohistochemistry has a relatively high quantitative sensitivity and produces a high signal-to-noise image under dark-field illumination; however, it exhibits poor photostability and is vulnerable to interference from tissue autofluorescence [18]. In contrast, PID staining, which was used in our study, produces an image with a high signal-to-noise ratio, even in the presence of tissue autofluorescence, owing to its brightness and photostability. Furthermore, the method used to process this type of image enables an automated calculation of the number of PIDs on the image, which corresponds to the expression levels of the target protein. Previous evidence has suggested that this method may dramatically improve the diagnostic capability of various targeted drug therapies [18,19]. The advantages of this novel technique have enhanced the credibility of our findings.

Limitations exist in this study. Its observational nature precludes the determination of a causal association between high expression levels of CSF1R in TAMs and mortality in never-smokers’ lung adenocarcinoma. The lack of a standardized evaluation method for CSF1R expression is another drawback. Nonetheless, we employed quantitative PID staining to precisely assess CSF1R expression in TAMs to minimize biases resulting from the subjective evaluation and susceptible intensities observed in DAB and fluorescent immunohistochemical staining [18,46]. Finally, this study may not be generalizable globally, because only Japanese patients at a single cancer hospital were enrolled. Therefore, our results must be validated in independent datasets.

## 5. Conclusion

In conclusion, the current study demonstrates that high expression levels of CSF1R in TAMs are associated with high mortality and that this prognostic association is stronger in never-smoking patients with lung adenocarcinoma than in ever-smoking patients. CSF1R-expressing TAMs may exert stronger tumor-promoting immunity in never-smoking patients than in ever-smoking patients; therefore, never-smokers with lung adenocarcinoma may be particularly responsive to CSF1R-targeted therapies. Given the growing popularity of immunotherapies [47,48,49], our findings, if validated, suggest the promising application of CSF1R-expressing TAMs as a biomarker or therapeutic target for the treatment of lung adenocarcinoma.

## Figures and Tables

**Table 1:** Clinicopathological and molecular characteristics of lung adenocarcinoma according to CSF1R expression status in tumor-associated macrophages.

Variables	*N* of Samples (%)	CSF1R Expression		
		Low–Moderate 130 (67%)	High 65 (33%)	*p* Values
Age (years)				0.40
<60	73 (37%)	46 (35%)	27 (42%)	
≥60	122 (63%)	84 (65%)	38 (58%)	
Gender				0.92
Male	106 (54%)	71 (55%)	35 (54%)	
Female	89 (47%)	59 (45%)	30 (46%)	
Smoking status				0.54
Never smoker	84 (43%)	54 (42%)	30 (46%)	
Ever smoker	111 (57%)	76 (58%)	35 (54%)	
Pathological stage				0.68
I	112 (57%)	76 (58%)	36 (55%)	
II–IV	83 (43%)	54 (42%)	29 (45%)	
Tumor differentiation				0.012
Well	84 (43%)	64 (50%)	20 (31%)	
Moderate–poor	110 (57%)	65 (50%)	45 (69%)	
*EGFR* status				0.11
Wild-type	71 (54%)	54 (59%)	17 (44%)	
Mutant	60 (46%)	38 (41%)	22 (56%)	
*KRAS* status				0.22
Wild-type	114 (87%)	78 (85%)	36 (92%)	
Mutant	17 (13%)	14 (15%)	3 (7.7%)	
*ALK* rearrangement				0.43
Negative	187 (96%)	124 (95%)	64 (98%)	
Positive	7 (3.6%)	6 (4.6%)	1 (1.5%)	
Number of TAMs				0.83
Low–moderate	131 (67%)	88 (68%)	43 (66%)	
High	64 (33%)	42 (32%)	22 (34%)	

TAM, tumor-associated macrophage.

**Table 2:** CSF1R expression in tumor-associated macrophages and patient mortality * in lung adenocarcinoma, stratified by smoking status.

Patients and CSF1R Expression Status	Lung Cancer-Specific Mortality				Overall Mortality			
	Univariable Analysis		Multivariable Analysis **		Univariable Analysis		Multivariable Analysis **	
	HR (95% CI)	*p* Values	HR (95% CI)	*p* Values	HR (95% CI)	*p* Values	HR (95% CI)	*p* Values
All patients								
CSF1R: low–moderate expression (*N* = 130)	1 (referent)		1 (referent)		1 (referent)		1 (referent)	
CSF1R: high expression (*N* = 65)	1.61 (1.02–2.52)	0.043	1.32 (1.00–2.49)	0.048	1.31 (0.87–1.95)	0.19	1.09 (0.72–1.65)	0.68
Never-smoking patients								
CSF1R: low–moderate expression (*N* = 54)	1 (referent)		1 (referent)		1 (referent)		1 (referent)	
CSF1R: high expression (*N* = 30)	2.90 (1.41–6.11)	0.0041	2.66 (1.28–5.66)	0.0088	2.63 (1.37–5.09)	0.0038	2.21 (1.05–4.77)	0.037
Ever-smoking patients								
CSF1R: low–moderate expression (*N* = 76)	1 (referent)		1 (referent)		1 (referent)		1 (referent)	
CSF1R: high expression (*N* = 35)	1.11 (0.59–2.00)	0.73	1.21 (0.65–2.19)	0.54	0.84 (0.48–1.43)	0.53	0.84 (0.47–1.43)	0.52
*p* values for interaction ***		0.049		0.12		0.0078		0.062

* Cox proportional hazards regression models were used to calculate the HR and 95% CI. ** The multivariable model included age at surgery (<60 vs. ≥60 years), gender (male vs. female), smoking status (never- vs. ever-smoker), pathological stage (I vs. II−IV), tumor differentiation grade (well vs. moderate–poor), *EGFR* status (wild-type vs. mutant), *KRAS* status (wild-type vs. mutant), *ALK* rearrangement (negative vs. positive), and number of TAMs (low–moderate vs. high). A backward stepwise elimination with *p* equal to 0.05 as the threshold was performed to select variables for the final models. *** *p* values for interactions between CSF1R expression status and smoking status were assessed using the Wald test on the cross-product of the CSF1R expression status (low–moderate vs. high) and smoking status variables (never- vs. ever-smoker) in the Cox model. CI, confidence interval; HR, hazard ratio; TAM, tumor-associated macrophage.

**Table 3:** CSF1R expression in tumor-associated macrophages and female patient mortality * in lung adenocarcinoma, stratified by smoking status.

Patients and CSF1R Expression Status	Lung Cancer-Specific Mortality				Overall Mortality			
	Univariable Analysis		Multivariable Analysis **		Univariable Analysis		Multivariable Analysis **	
	HR (95% CI)	*p* Values	HR (95% CI)	*p* Values	HR (95% CI)	*p* Values	HR (95% CI)	*p* Values
Female patients								
CSF1R: low–moderate expression (*N* = 59)	1 (referent)		1 (referent)		1 (referent)		1 (referent)	
CSF1R: high expression (*N* = 30)	2.28 (1.07–4.88)	0.033	4.16 (1.62–11.0)	0.0034	2.23 (1.13–4.39)	0.021	2.37 (1.19–4.73)	0.015
Never-smoking female patients								
CSF1R: low–moderate expression (*N* = 48)	1 (referent)		1 (referent)		1 (referent)		1 (referent)	
CSF1R: high expression (*N* = 22)	2.78 (1.22–6.32)	0.015	3.31 (1.42–7.80)	0.0060	2.69 (1.30–5.53)	0.0081	3.18 (1.50–6.76)	0.0028
Ever-smoking female patients								
CSF1R: low–moderate expression (*N* = 11)	1 (referent)		1 (referent)		1 (referent)		1 (referent)	
CSF1R: high expression (*N* = 8)	1.33 (0.16–11.1)	0.77	0.92 (0.11–7.86)	0.93	1.33 (0.16–11.1)	0.77	0.92 (0.11–7.86)	0.93
*p* values for interaction ***		0.49		0.29		0.51		0.31

* Cox proportional hazards regression models were used to calculate the HR and 95% CI. ** The multivariable model included age at surgery (<60 vs. ≥60 years), gender (male vs. female), smoking status (never- vs. ever-smoker), pathological stage (I vs. II−IV), tumor differentiation grade (well vs. moderate–poor), *EGFR* status (wild-type vs. mutant), *KRAS* status (wild-type vs. mutant), *ALK* rearrangement (negative vs. positive), and number of TAMs (low–moderate vs. high). A backward stepwise elimination with *P* equal to 0.05 as the threshold was performed to select variables for the final models. *** *p* values for interactions between CSF1R expression status and smoking status were assessed using the Wald test on the cross-product of the CSF1R expression status (low–moderate vs. high) and smoking status variables (never- vs. ever-smoker) in the Cox model. CI, confidence interval; HR, hazard ratio; TAM, tumor-associated macrophage.

## Abbreviations

The following abbreviations are used in this manuscript:

BSA	bovine serum albumin
CI	confidence interval
DAB	diaminobenzidine
HR	hazard ratio
JFCR	Japanese Foundation for Cancer Research
PBS	phosphate-buffered saline
PID	phosphor-integrated dot
SNP	single nucleotide polymorphism
TAM	tumor-associated macrophage
